# Personalized Protein Supplementation Improves Total Protein, Leucine, and Energy Intake in (Pre)Sarcopenic Community-Dwelling Older Adults in the ENHANce RCT

**DOI:** 10.3389/fnut.2021.672971

**Published:** 2021-08-09

**Authors:** Lenore Dedeyne, Jolan Dupont, Sabine Verschueren, Katrien Koppo, Jos Tournoy, Christophe Matthys, Evelien Gielen

**Affiliations:** ^1^Gerontology and Geriatrics, Department of Public Health and Primary Care, Katholieke Universiteit Leuven, Leuven, Belgium; ^2^Department of Geriatric Medicine, UZ Leuven, Leuven, Belgium; ^3^Department of Rehabilitation Sciences, Katholieke Universiteit Leuven, Leuven, Belgium; ^4^Exercise Physiology Research Group, Department of Movement Sciences, Katholieke Universiteit Leuven, Leuven, Belgium; ^5^Clinical and Experimental endocrinology, Department of Chronic Diseases, Metabolism and Ageing (CHROMETA), Katholieke Universiteit Leuven, Leuven, Belgium; ^6^Department of Endocrinology, UZ Leuven, Leuven, Belgium

**Keywords:** protein, leucine, supplement, sarcopenia, geriatrics, age, energy

## Abstract

Recommendations concerning protein quantity, source, and leucine intake for older adults are difficult to reach by regular dietary intake. This randomized clinical trial assesses in sarcopenic community-dwelling older adults (i) the regular (non-supplemented) daily protein and leucine intake; and (ii) the effect of personalized protein supplementation (aiming for an evenly distributed total protein intake of 1.5 g·kg^−1^·d^−1^ of body mass, accounting for energy intake) on regular and total (dietary and supplemental) intake. A preliminary feasibility study in participants of the ongoing Exercise and Nutrition for Healthy AgeiNg (ENHANce) study was performed with the objective to assess the intake and distribution of regular dietary protein and leucine, protein source and energy intake in (pre)sarcopenic community-dwelling older adults. Moreover, this study aimed to assess if personalized protein supplementation was feasible without negatively affecting regular dietary intake. ENHANce (NCT03649698) is a 5-armed RCT that assesses the effect of anabolic interventions on physical performance in (pre)sarcopenic older adults. In August 2019, *n* = 51 participants were included in ENHANce with complete available data on dietary intake at screening and thus eligible for inclusion in present analysis. Of these, *n* = 35 participants completed the intervention period of ENHANce at the moment of analysis, allowing an exploration of the effect of supplementation on regular dietary intake. The regular dietary protein intake of 51 (pre)sarcopenic adults (73.6 ± 6.5 years) was 1.06 ± 0.3 g·kg^−1^·d^−1^ of body mass. Protein supplementation (*n* = 20) improved total protein intake to 1.55 ± 0.3 g·kg^−1^·d^−1^ of body mass (*P* < 0.001) without affecting regular dietary protein (*P* = 0.176) or energy intake (*P* = 0.167). Placebo supplementation (*n* = 15) did not affect regular dietary protein intake (*P* = 0.910) but decreased regular dietary energy intake (*P* = 0.047). Regular leucine intake was unevenly distributed over the day, but increased by supplementation at breakfast (*P* < 0.001) and dinner (*P* = 0.010) to at least 2.46 g leucine·meal^−1^, without reducing regular dietary leucine intake (*P* = 0.103). Animal-based protein intake—the main protein source—was not affected by supplementation (*P* = 0.358). Personalized protein supplementation ensured an adequate quantity and even distribution of protein and leucine over the day, without affecting regular dietary protein or energy intake.

## Introduction

Sarcopenia is the loss of skeletal muscle function and mass through aging (1, 2). Exercise is the primary treatment component and improves older adults' muscle strength, muscle mass and physical functioning (2). The combination of exercise with protein supplementation results in even greater gains in lean mass and leg strength compared to exercise alone (3, 4). Leucine plays a dual role as regulator of intracellular signaling pathways leading to protein synthesis and as substrate for protein synthesis (5, 6). Indeed, sarcopenia has been associated with low protein intake and specific dietary patterns (butter, red meat, gravy and potato) (7, 8). An umbrella review recommends supplementation of leucine for older adults with sarcopenia to improve muscle mass (4).

Although it is clear that protein supplementation is of importance in the treatment of sarcopenia, multiple variables of protein supplementation (quantity, quality, and distribution) must be considered. First, the muscle protein synthesis (MPS) in response to exercise and protein intake is blunted in older adults compared to younger individuals (9). As a result, the Recommended Dietary Allowance (RDA) for protein for adults (0.8 g·kg^−1^·d^−1^ of body mass) is insufficient for older adults (10–14). Therefore, expert groups and guidelines recommend a protein intake of 1.0 g·kg^−1^·d^−1^ of body mass for older adults and up to 1.5 g·kg^−1^·d^−1^ of body mass in case of acute or chronic diseases (15–17). Second, the protein quality is relevant for sufficient anabolic stimulation of MPS (16). Protein quality is determined by the protein source (plant- or animal-based) that influences the digestibility and the amino-acid composition (e.g., essential amino-acids such as leucine). Third, an even protein distribution over the day is recommended. More specifically, the PROT-AGE group recommends for healthy older adults 25 g protein per meal, of which 2.5–2.8 g of leucine. This equal distribution needs to ensure sufficient protein intake throughout the day to maximize stimulation of MPS (16).

Recent studies have described regular dietary protein intake and its distribution over the day in sarcopenic older adults (18, 19). However, we are not aware of studies that have described the intake of leucine and its distribution over the day in this population.

The first aim of this study is to assess the intake and distribution of regular dietary protein (primary outcome) and leucine, the protein source and energy intake in (pre)sarcopenic community-dwelling older adults. The supplementation of participants with protein supplements may affect regular dietary intake. In that regard, our second aim was to assess if personalized protein supplementation could ensure an adequate protein intake in older adults with (pre)sarcopenia and if this supplementation affected the dietary protein, leucine or energy intake.

We hypothesized that (pre)sarcopenic community-dwelling older adults do not reach the recommended dietary protein intake through their regular dietary intake. Also, we hypothesize that that through personalized protein supplementation the recommendation of 1.5 g·kg^−1^·d^−1^ of body mass can be reached without affecting regular dietary protein or energy intake.

## Methods

### Study Design

A preliminary feasibility study in participants of the ongoing Exercise and Nutrition for Healthy AgeiNg (ENHANce) study was performed with participants included in a period between February 2018 and August 2019. The food diaries of (pre)sarcopenic participants that were screened for the ENHANce study were included in the analysis to describe regular protein intake (*n* = 51). For analysis of the effect of the protein supplement on regular dietary intake, participants were divided in two groups: the protein supplement group (arm 2, 3, and 4 of ENHANce) or the placebo supplement group (arm 1 and 5 of ENHANce). In total, *n* = 35 ENHANce participants initiated and completed the ENHANce intervention period as well as a second food diary after 12 weeks of the ENHANce study. These participants were included in the analyses to describe the effect of personalized supplementation on regular protein intake.

The ENHANce study is an ongoing, single-center, placebo-controlled triple blinded randomized controlled trial, that was initiated in February 2018. Details of the methods are described elsewhere (20). In brief, the ENHANce study aims to examine the effect of combined anabolic interventions (exercise and food compounds) on physical functioning in (pre)sarcopenic older adults. The ENHANce study consists of a screening visit (1 month before the start of intervention) and a 12-week intervention period. During the screening visit, fulfillment with the inclusion criteria was checked, participants were asked to complete a food diary during the first week after screening and participants were block randomized (based on gender) into one of the five intervention arms: (1) Exercise intervention + protein placebo + PUFA's placebo; (2) Protein + PUFA's placebo; (3) Exercise intervention + protein + PUFA's placebo; (4) Exercise intervention + protein + PUFA's; (5) Control group: protein placebo + PUFA's placebo. The 12-week intervention period started and ended with an extensive visit including measurements of physical and cognitive abilities (Supplementary Figure 1). The study was approved by KU Leuven/UZ Leuven Ethics Committee (s60763) and registered on ClinicalTrials.gov (NCT03649698).

### Study Population and Inclusion Criteria

Participants of present study are community-dwelling older adults (aged ≥ 65 years) living in Belgium. Participants were recruited from Leuven and surrounding area in a number of different ways, including senior citizen organizations, as described in the study protocol (20). The ENHANce participants screened between February 2018 and July 2019 had “presarcopenia,” “sarcopenia,” or “severe sarcopenia” (in this article also referred to as “sarcopenia”) according to the operational definition of the European Working Group on Sarcopenia in Older People (EWGSOP1) (1). From November 2018 onwards, participants had “probable sarcopenia,” “confirmed sarcopenia,” or “severe sarcopenia” (in this article referred to as “sarcopenia”) according to EWGSOP2 (2). The period between November 2018 and July 2019 was a transition period where both definitions were accepted. Other inclusion criteria were: to be able to communicate in Dutch, English or French, no allergy to milk, soy, peanut or peanut oil, Mini-Mental State Examination (MMSE) > 21 (21), no terminal illness with a prognosis <6 months, protein intake lower than 1.5 g·kg^−1^·d^−1^ of body mass 1 week after screening, not following a training program equal to or more than twice per week during the last 6 months, no uncontrolled disease or acute cardiovascular problems (no medical contraindication to perform physical activities), glomerular filtration rate > 30 ml ·min^−1^·(1.73 m^2^)^−1^, fasting glycaemia < 126 mg ·dl^−1^ and no treatment for diabetes and no impairments or diseases that impose problems to study participation according to the investigators. Written informed consent was obtained by the participant before the start of the screening visit.

### Physical Assessment and Sarcopenia Assessment

Participant characteristics including age (years), body mass index (BMI, kg/m^2^), and risk of malnutrition were assessed. Body weight (SECA, model no 8801321009, SECA UK Ltd.) to the nearest 0.1 kg and height (Harpenden stadiometer, Holtain Ltd., Crosswell, UK) to the nearest 0.1 cm, were measured standing upright, barefoot and in light clothing. The BMI was calculated as weight/height^2^. The Mini Nutritional Assessment—Short Form (MNA-SF) was completed, classifying participants in a range between 0 and 14 points. A score of 0–7 indicates malnourishment and a score of 8–11 identifies persons at risk of malnutrition (22).

Appendicular lean mass was assessed by dual-energy X-ray absorptiometry (DXA, Horizon A scanner, Hologic Inc., Bedford, MA, USA). Low muscle mass was defined by the skeletal muscle index (SMI), which is the appendicular lean mass (kg) divided by height^2^ (m^2^). Participants with presarcopenia and sarcopenia (EWGSOP1) and confirmed or severe sarcopenia (EWGSOP2) had low muscle mass. The cut-off of low SMI in EWGSOP1 was SMI < 7.26 kg/m^2^ for men and SMI < 5.50 kg/m^2^ for women (1) and in EWGSOP2, the cut-off was SMI < 7.00 kg/m^2^ for men and SMI < 5.50 kg/m^2^ for women (2).

Handgrip strength was measured by hand dynamometry (Jamar 1, TEC Inc., Clifton, NJ, USA) while the participant was seated with the elbow at a 90° angle according the American Society of Hand Therapists (23) for EWGSOP1 measurements. The mean value of three consecutive measurements of the dominant hand [according to Fried et al. (24)] was recorded for scoring purposes. According to the EWGSOP1 criteria, the cut-off values were dependent of the BMI (1). The EWGSOP2 proposed the Southampton protocol to assess handgrip strength six times, alternating sides and the maximal value was reported (25). According to the EWGSOP2, low grip strength was defined as < 16 kg for women and < 27 kg for men according to the EWGSOP2 (2).

Gait speed was assessed over a distance of six meters. Participants were instructed to walk 6 m on usual pace (walking aids allowed). The time was measured over 4 m: from the moment the participant's foot passed the mark of the 1 m line on the ground until the foot passed the mark of the 5 m line, according to the BC guidelines (26). The mean of three tests was calculated for scoring purposes. A cut-off of ≤ 0.8 m·s^−1^ was used to define low gait speed according to the definition of the EWGSOP (1) and EWGSOP2 (2).

The chair stand test was performed by measuring the time required to rise five consecutive times as quickly as possible from a chair with arms folded in front of the chest (27). The cut-off point for low physical performance was >15 s, as defined according to the EWGSOP2 (2).

### Estimated Dietary Records

Participants completed a 4-day estimated dietary records (EDR) on non-consecutive days including 3 week- and 1 weekend day. Based on the formula of Beaton (28) including the within-subject standard deviation of older (65–85 years) adults living in the north of England (29), a 4-day EDR is appropriate to assess protein intake of an individual. Non-consecutive days and alternating periods were chosen to obtain a better estimate of the day-to-day variability (30). At screening, recorded days were Monday, Wednesday, Friday, Sunday, and at week 12, recorded days were Tuesday, Thursday, Saturday, and Monday. Participants were instructed to meticulously record food and beverage intake for 24 h. Portion sizes were documented in pre-defined household units and determined based on “Maten en Gewichten,” a guide to convert qualitative data relating to the amount (estimates) of food ingested into quantitative data in a uniform manner (31). Time of day, description of food and brand of food were documented in the EDR. Quality of records was assured by reviewing records together with the participant in order to complete the records as accurately as possible.

### Assessment of Dietary Intake

Dietary intake was computed based on the Belgian and USA Food Composition Table [Nubel, BE (31) and USDA FoodData Central, USA] (32). The estimate of mean protein (g·kg^−1^·d^−1^ of body mass, g·day^−1^ or % of total energy·day^−1^), fat (% of total energy·day^−1^), carbohydrates (% of total energy·day^−1^), energy (kcal·kg^−1^·d^−1^ of body mass or kcal·day^−1^), leucine (g·day^−1^or mg·kg^−1^·d^−1^ of body mass) consumption was calculated as a mean of 4 days. The actual body weight of the participant was used. Snacks were defined as the eating moments between meals. Snack 1 is defined as snacks between breakfast and lunch, snack 2 between lunch and dinner and snack 3 after dinner, before bedtime. Factors of protein quality included the protein source and amino-acids composition, such as leucine. The protein source (plant/animal source) of each dietary protein product was based on the Dutch Food Composition Database (33). Amino-acid composition was based on the USDA food composition database (USDA FoodData Central) (32). The amino-acid composition of a food item was analyzed when the relative protein contribution of this product was 1% or more of the total protein intake per day. Regular dietary intake reflects the protein intake originating from foods while the total intake reflects the dietary intake and the intake from supplements.

### Calculation of Protein Supplementation

The aim of the personalized supplementation was to reach the before mentioned protein recommendation for older adults with acute or chronic diseases of 1.5 g·kg^−1^·d^−1^ of body mass (16). Meals were not substituted with a supplement, but a supplement was added to a meal if necessary. The exact amount (to the nearest 0.1 g·kg^−1^·d^−1^ of body mass) of protein needed to reach this recommendation was calculated. Each meal, if needed, was supplemented according to the following rule: a combined (diet + supplement) protein intake of at least 25 g protein/meal (breakfast/lunch/dinner) must be reached. Next, in case the protein intake of 1.5 g·kg^−1^·d^−1^ of body mass intake per day was not yet reached, the combined (diet + supplement) protein intake per meal was equally increased over the meals (breakfast/lunch/dinner) up to a maximum of 35 g protein·meal^−1^, but a maximum of 700 kcal·meal^−1^ is never exceeded [35% of average requirement for energy intake for older adults (34)]. In case the protein intake of 1.5 g·kg^−1^·d^−1^ of body mass was not yet reached after applying the above rules, the snack before bedtime (snack 3) was supplemented. In summary, if supplementation was needed, at least one evening snack and maximum three main meals and the evening snack were enriched. For supplementation calculations, the body weight of participants with a BMI < 22 or >27 kg/m^2^ was adjusted: the adjusted body weight of participant with BMI < 22 was the body weight that would result in a BMI of 22 kg/m^2^, the adjusted body weight of participant with BMI > 27 was the body weight that would result in a BMI of 27 kg/m^2^ (35). The supplemented protein (g) was visualized for each individual receiving the protein supplement in Supplementary Figure 2.

### Participant Instructions for Supplement Intake

Participants were instructed to initiate the intake of the powder supplement (with protein or placebo powder) 5 days before the start of the 12-week intervention period. Participants received instructions on how to dissolve the protein powder and suggestions of drinks and foods to mix with the powder before consumption. For that purpose, each participant received an individualized drinking glass for each meal or snack that had to be supplemented. By means of a line on this drinking glass, the amount of powder to be taken with this meal/snack was indicated. For each meal/snack at home, the participant completed the glass for that meal/snack up to the marked line with the provided powder. Participants in the protein supplement group received Resource Instant Protein (Nestlé, 4.5 g protein per 5 g powder, 9.14% leucine). The placebo product was Resource dextrin maltose (Nestlé, 0 g protein). Nutritional content of both powders is described in Supplementary Table 1. The powder boxes and drinking glasses were prepared by an unblinded researcher, who did not participate in study visits or had contact with the participants. The boxes for protein or placebo powder products were equal looking blank boxes.

### Assessment of Compliance

Compliance with powder supplement intake was assessed by weighing the amount of powder that was taken from the provided powder box at each study visit (seven times in 12-week period). The compliance was defined as the proportion of amount of powder that was actually removed from the boxes to the prescribed amount of powder that was suggested to be taken.

### Statistics

#### Sample Size

The data reporting in the current study is compliant with the STROBE-nut guidelines (36). As mentioned in the methods section, these analyses are based on data from the ongoing ENHANce study. Although the ENHANce study is primarily powered on functional outcomes, the number of participants needed to detect a difference of 0.5 g·kg^−1^·d^−1^ of body mass with a standard deviation of 0.22, two time points and an assumed correlation of 0.9, is three participants per group with a power of 0.80 with alpha level of 0.05. Therefore, these analyses are sufficiently powered to detect differences between the different time points (visits). *Post-hoc* power analyses are therefore not useful.

#### Statistical Analyses

Data analyses were performed using IBM SPSS Statistical package (IBM Corp. Released 2017 IBM SPSS Statistics for Windows, Version 25.0, Armonk, NY). Normal continuous variables are presented as the mean and standard deviation (SD), while the non-normal continuous variables are presented as the median with interquartile ranges (IQR). Normality of variables was checked using a Shapiro–Wilk test. To compare differences in normal distributed variables between regular dietary intake at screening or wk 12 or total intake at wk 12, repeated measures ANOVA with *post-hoc* paired sample *t*-test were used. For non-normal distributed variables were Friedman test (non-parametric alternative to the repeated measures ANOVA) with *post-hoc* Wilcoxon signed-rank tests used. For comparisons of variables between regular dietary intake at screening or wk 12, the paired *t*-test or Wilcoxon signed-rank test was used depending on distribution. A *p*-value of < 0.05 (two-tailed) was considered statistically significant.

## Results

### Participant Characteristics

**Figure 1:**
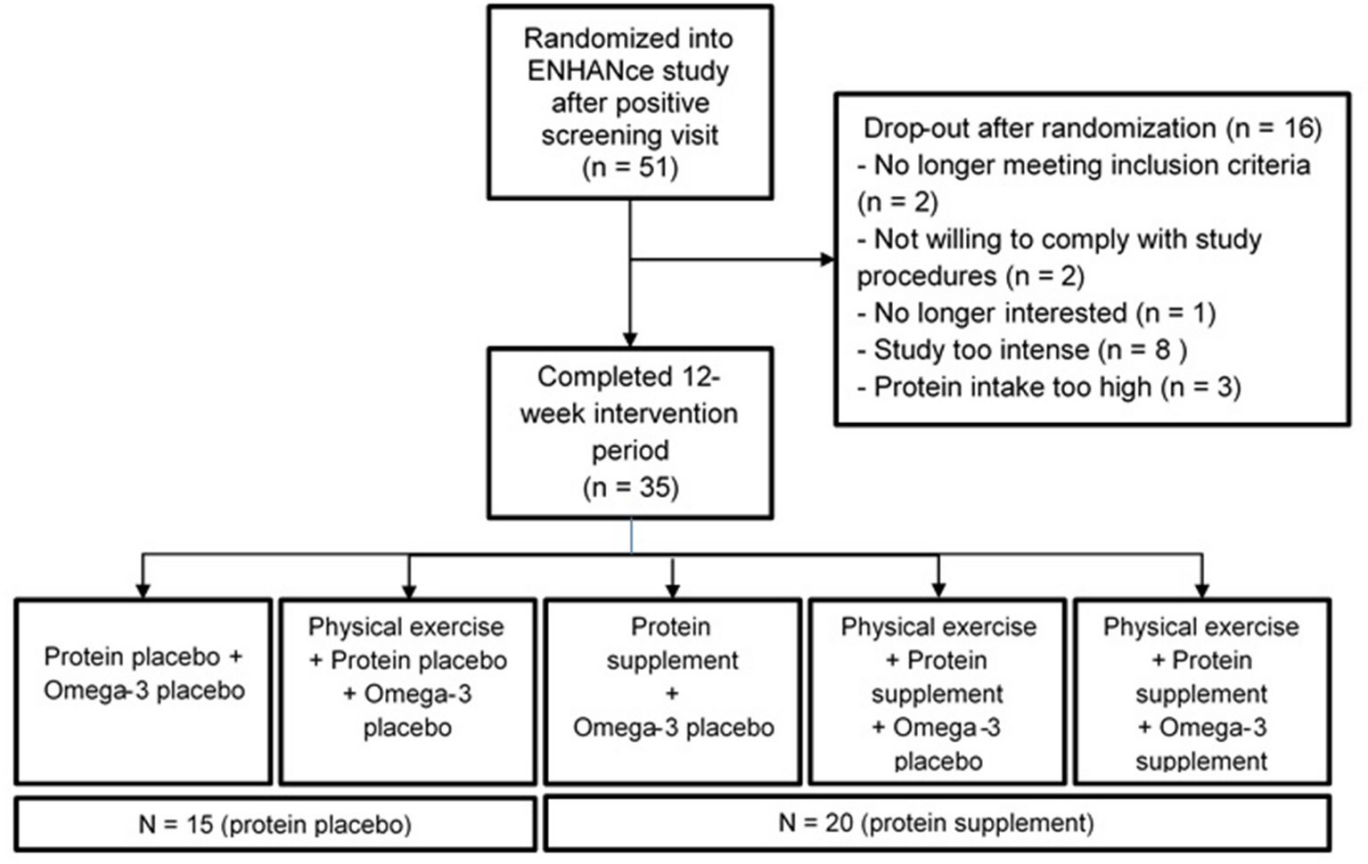
Overview of flow of participant of the ENHANce study relevant for this article.

Fifty-one participants in the ENHANce study completed the screening visit and EDR at screening. Thirty-five participants completing EDR at wk 12 since 16 participants dropped out before completion of the 12-week study period. Fifty percent (*n* = 8) of the dropouts perceived the study as “too intense,” 19% (*n* = 3) had a protein intake above 1.5 g g·kg^−1^·d^−1^ of body mass, two participants were no longer meeting inclusion criteria, two participants were not willing to comply with study procedures, and one participant was no longer interested in study participation. Twenty of the thirty-five participants received a protein supplement, 15 received a placebo supplement (Figure 1). Study participants at screening visit were 73.6 years old (± 6.5) and 52.9% were female. Mean total fat and total carbohydrate intake were, respectively 36.3 ± 7.6% of total energy·day^−1^ and 42.5 ± 9.2% of total energy·day^−1^ (Table 1). Forty-four participants were included according to EWGSOP1 of which 39 participants were presarcopenic and five participants were sarcopenic; seven participants were according to EWGSOP2, one probable sarcopenic and six confirmed sarcopenic.

**Table 1:** Characteristics (mean ± SD) of community-dwelling (pre)sarcopenic older adults at start of the intervention period.

	***N***	**All sarcopenic participants**	**Intervention type**	
			**Protein (*n* = 20)**	**Placebo (*n* = 15)**
Age (years)	51	73.6 ± 6.47	73.9 ± 6.32	74.9 ± 8.03
Female (%)	51	52.9	50	40
BMI (kg/m^2^)	51	24.2 ± 4.03	23.4 ± 2.40	25.3 ± 2.49
Gait speed (m·s^−1^)	51	1.16 ± 0.28	1.11 ± 0.21	1.09 ± 0.13
**Handgrip strength (kg)**				
Females	27	24.3 ± 4.76	24.5 ± 4.86	6: 25.8 ± 3.05
Males	24	37.0 ± 9.54	39.7 ± 7.38	9: 40.1 ± 7.60
**SMI (kg/m** ^**2**^ **)**				
Females	26	5.18 ± 0.36	5.11 ± 0.46	6: 5.37 ± 0.21
Males	24	6.60 ± 0.49	6.43 ± 0.37	9: 6.85 ± 0.37
Chair stand test (s)	18	14.2 ± 4.77	15.0 ± 4.97	13.1 ± 2.29
MNA-SF (normal/at risk/malnourished)	36	31/5/0	17/2/0	13/2/0
Total fat intake (% of total energy)	51	36.3 ± 7.61	35.0 ± 8.51	36.3 ± 7.50
Total carbohydrates intake (% of total energy)	51	42.5 ± 9.15	45.5 ± 9.45	39.6 ± 10.1
Total protein intake (g·kg^−1^·d^−1^ of body mass)	51	1.06 ± 0.26	1.04 ± 0.22	1.01 ± 0.21

*Values presented as mean ± SD. N, number of included subjects; BMI, body mass index; SMI, skeletal muscle index; MNA-SF, mini nutritional assessment—short form*.

### Energy Intake

The mean regular dietary energy intake at screening was 2,000 kcal·day^−1^, or 29.3 kcal·kg^−1^·d^−1^ of body mass (*n* = 51). In the protein supplement group, supplementation significantly increased the total energy intake (2,110 kcal·day^−1^, 30.4 kcal·kg^−1^·d^−1^ of body mass) compared to the regular dietary intake at wk 12 (1,990 kcal·day^−1^, 28.9 kcal·kg^−1^·d^−1^ of body mass). In the placebo supplement group, regular dietary energy intake (kcal·day^−1^) did not significantly change, except that the regular dietary intake decreased from screening (29.1 kcal·kg^−1^·d^−1^ of body mass) to wk 12 (26.3 kcal·kg^−1^·d^−1^ of body mass; *p* = 0.047; Table 2).

**Table 2:** Energy intake (median and IQR) based on the 4-day EDR of subgroup of community-dwelling (pre)sarcopenic older adults receiving protein supplement (*n* = 20) or placebo supplement (*n* = 15).

	**Energy**	**Dietary intake, screening**	**Dietary intake, wk 12**	**Total intake, wk 12**	***P*** **-value**
**All sarcopenic participants** (***n*** **= 51)**	**kcal·d** ^**−1**^	2,000 ± 414			
	**kcal·kg** ^**−1**^ **·d** ^**−1**^ **of body mass**	29.3 ± 6.3			
**Protein (** ***n*** **= 20)**	**kcal·d** ^**−1**^	1,830 (1,660–2,000)	1,990 (1,600–2,170)	2,110 (1,780–2,320)	0.067 (D_s_ vs. T_wk12_); 0.502 (D_wk12_ vs. D_s_); <0.001^*^ (D_wk12_ vs. T_wk12_)
	**kcal·kg** ^**−1**^ **·d** ^**−1**^ **of body mass**	30.1 (23.4–37.7)	28.92 (24.5–31.8)	30.37 (25.8–33.0)	0.681 (D_s_ vs. T_wk12_); 0.167 (D_wk12_ vs. D_s_); <0.001^*^ (D_wk12_ vs. T_wk12_)
**Placebo** (***n*** **= 15)**	**kcal·d** ^**−1**^	2,000 ± 326	2,020 ± 434		0.873 (D_s_ vs. D_wk12_)
	**kcal·kg** ^**−1**^ **·d** ^**−1**^ **of body mass**	29.1 (26.5–65.7)	26.28 (23.0–31.7)		0.047 (D_s_ vs. D_wk12_)

D_s_, dietary intake, screening. D_wk12_, dietary intake, wk 12. T_wk12_, total intake at week 12. P-value repeated measures ANOVA between visits, dietary intake (kcal·d^−1^): 0.001^*^,

dietary intake (kcal·kg^−1^·d^−1^ of body mass): 0.004^*^.

*Bold p-values represent statistically significant results (p < 0.05)*.

### Protein Quantity

The mean regular dietary protein intake at screening was 72.8 ± 17.6 g·day^−1^, or 1.06 ± 0.3 g·kg^−1^·d^−1^ of body mass (*n* = 51). Participants receiving a protein supplement (*n* = 20) showed an increased total protein intake (105 g·day^−1^, 1.55 ± 0.3 g·kg^−1^·d^−1^ of body mass) compared to regular dietary intake at screening (69.1 g·day^−1^, 1.04 ± 0.2 g·kg^−1^·d^−1^ of body mass) or wk 12 (74.7 g·day^−1^, 1.13 ± 0.4 g·kg^−1^·d^−1^ of body mass), whilst participants receiving a placebo supplement (*n* = 15) did not significantly change regular dietary protein intake (g·d^−1^ or g·kg^−1^·d^−1^ of body mass). Compared to regular dietary intake at screening (15.8 energy%) or wk 12 (15.8 energy%), protein supplementation resulted in an increased total relative protein intake (20.9 energy%; Table 3).

**Table 3:** Protein intake (mean ± SD) based on the four-day EDR of all community-dwelling (pre)sarcopenic older adults and subgroups of *n* = 20 receiving protein supplement and *n* = 15 receiving placebo supplements.

	**Protein**	**Dietary intake, screening**	**Dietary intake, wk 12**	**Total intake, wk 12**	***P*** **-value**
**All sarcopenic participants (***n*****=** 51)**	**g·d** ^**−1**^	72.8 ± 17.6			
	**g·kg** ^**−1**^ **·d** ^**−1**^ **of body mass**	1.06 ± 0.26			
**Protein (***n*****=** 20)**	**g·d** ^**−1**^	69.1 ± 10.7	74.7 ± 18.5	104.7 ± 22.0	<0.001^*^ (D_s_ vs. T_wk12_); 0.149 (D_wk12_ vs. D_s_); <0.001^*^ (D_wk12_ vs. T_wk12_)
	**g·kg** ^**−1**^ **·d** ^**−1**^ **of body mass**	1.04 ± 0.22	1.13 ± 0.36	1.55 ± 0.26	<0.001^*^ (D_s_ vs. T_wk12_); 0.176 (D_wk12_ vs. D_s_); <0.001^*^ (D_wk12_ vs. T_wk12_)
	**% of total energy**	15.5 ± 2.98	15.8 ± 2.59	19.4 ± 3.30	<0.001^*^ (D_s_ vs. T_wk12_); 0.625 (D_wk12_ vs. D_s_); <0.001^*^ (D_wk12_ vs. T_wk12_)
**Placebo (***n*****=** 15)**	**g·d** ^**−1**^	73.8 ± 18.6	74.9 ± 15.1		0.862 (D_s_ vs. D_wk12_)
	**g·kg** ^**−1**^ **·d** ^**−1**^ **of body mass**	1.00 (0.80 – 1.20)	1.02 (0.87–1.17)		0.910 (D_s_ vs. D_wk12_)

D_s_, dietary intake, screening. D_wk12_, dietary intake at wk 12. T_wk12_, total intake at wk 12. P-value repeated measures ANOVA between visits, dietary intake (g·d^−1^): < 0.001^*^,

dietary intake (kcal·kg^−1^·d^−1^ of body mass): < 0.001^*^,

dietary intake (% of total energy): < 0.001^*^.

*Bold p-values represent statistically significant results (p < 0.05)*.

**Figure 2:**
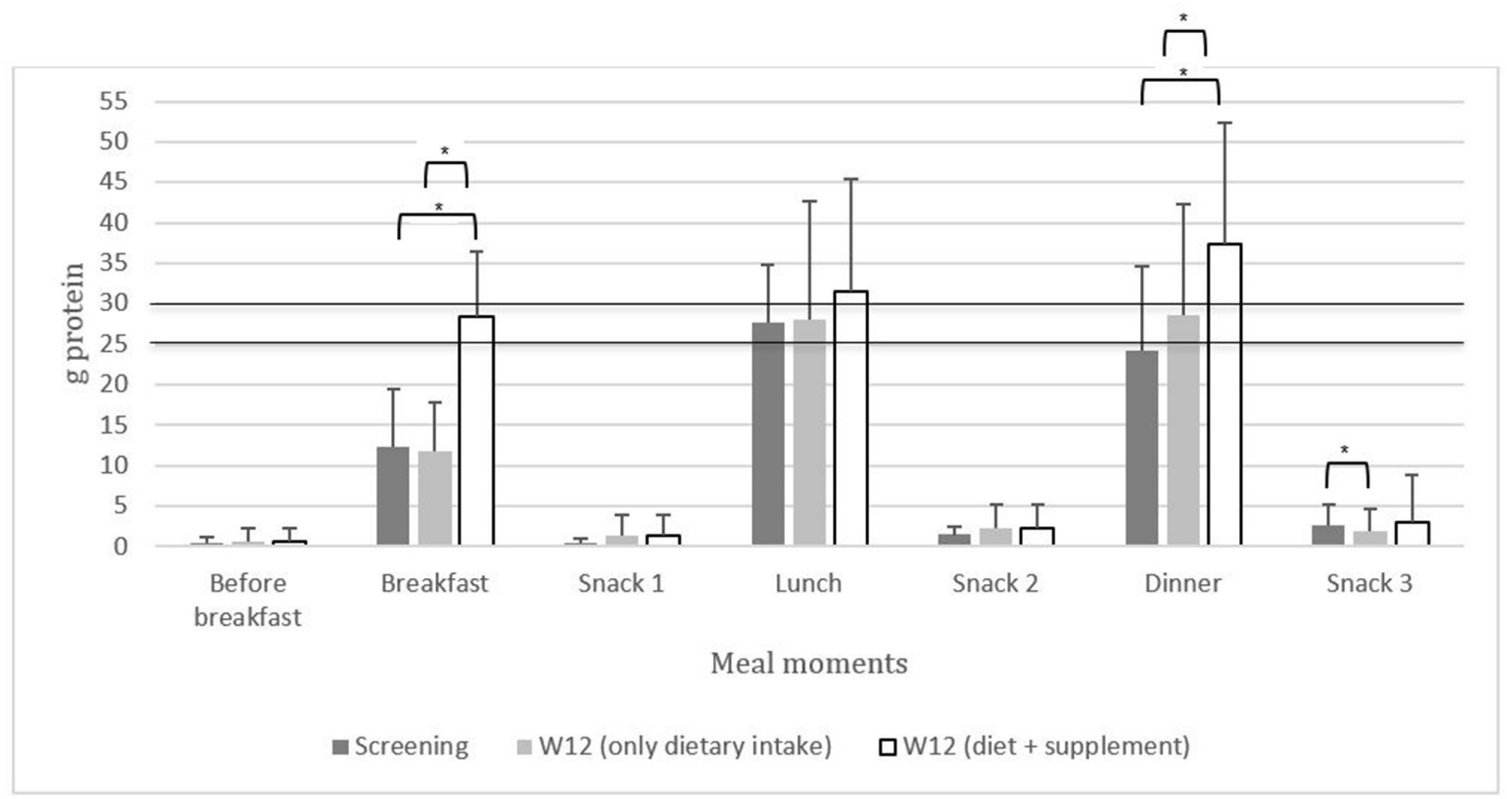
The distribution of protein intake [g (mean ± SD)] across breakfast, lunch, dinner, and snack times in community-dwelling (pre)sarcopenic older adults. The line of 25 and 30 g represents the amount of protein required per meal to maximally stimulate muscle protein synthesis according to the PROT-AGE Study Group (16). *p < 0.05.

At screening, the regular dietary distribution of protein intake at breakfast, lunch, dinner, and snack times was unequal, and breakfast did not meet the 25 g protein per meal threshold (*n* = 51, Supplementary Table 2). Protein supplementation resulted in higher total protein intake (28.2 g at breakfast *p* < 0.001; 36.7 g at dinner, *p* = 0.004) compared to regular dietary intake at screening and wk 12 at breakfast (11.7 g, *p* < 0.001; 10.2 g, *p* < 0.001) and dinner (24.4 g, *p* = 0.002; 26.2 g, *p* = 0.001). Dietary protein intake after dinner/before bedtime (snack 3) decreased (*p* = 0.023) at wk 12 compared to screening (Figure 2; Supplementary Table 2).

### Protein Quality

The contribution of animal- and plant-based sources to daily protein intake was not significantly different between regular dietary intake at screening (animal 63.5%; plant 36.5%) and wk 12 (animal 61.9%, plant 38.1%; *p* = 0.358). The daily intake of the individual amino acids (g) did not significantly change between regular dietary intake at screening and wk 12, except a slight increase in cysteine intake (Supplementary Table 3). Daily dietary leucine intake at screening (6.1 ± 1.0 g·day^−1^, 114 ± 57 mg·kg^−1^·d^−1^ of body mass) or wk 12 (6.8 ± 1.6 g·day^−1^, 102 ± 30 mg·kg^−1^·d^−1^ of body mass) increased by supplementation (9.5 ± 2.2 g·day^−1^, 140 ± 24 mg·kg^−1^·d^−1^ of body mass), without affecting regular dietary leucine intake (Table 4).

**Table 4:** Leucine intake (g·d^−1^) or (mg·kg^−1^·d^−1^ of body mass) mean ± SD and distribution of leucine intake (median and interquartile range) of a subgroup of 20 community-dwelling (pre)sarcopenic older adults receiving protein supplement (*n* = 20).

	**Dietary intake, screening**	**Dietary intake**, **wk 12**	**Total intake**, **wk 12**	***P*** **-value**			
**g·d** ^**−1**^	6.07 ± 0.95	6.75 ± 1.64	9.50 ± 2.15	<0.001^*^ (D_s_ vs. T_wk12_); 0.103 (D_wk12_ vs. D_s_); <0.001^*^ (D_wk12_ vs. T_wk12_)			
**mg·kg** ^**−1**^ **·d** ^**−1**^ **of body mass**	114 ± 57.3	102 ± 30.3	140 ± 23.8	0.041^*^ (D_s_ vs. T_wk12_); 0.348 (D_wk12_ vs. D_s_); <0.001^*^ (D_wk12_ vs. T_wk12_)			
	**Before breakfast**	**Breakfast**	**Snack 1**	**Lunch**	**Snack 2**	**Dinner**	**Snack 3**
**Dietary intake, screening (g·meal** ^**−1**^ **)**	0.00 (0.00–0.01)	0.87 (0.49–1.09)	0.01 (0.00–0.08)	2.34 (2.05–2.88)	0.15 (0.06–0.22)	1.99 (1.17–2.66)	0.21 (0.01–0.59)
**Dietary intake, wk 12 (g·meal** ^**−1**^ **)**	0.00 (0.00–0.01)	0.90 (0.43–1.40)	0.04 (0.00–0.14)	2.55 (1.80–3.08)	0.14 (0.04–0.27)	2.35 (1.22–3.54)	0.07 (0.00–0.51)
**Total intake, wk 12 (g·meal^−1^)**	0.00 (0.00–0.01)	2.46 (1.89–3.24)^°^^#^	0.04 (0.00–0.14)	2.81 (2.30–3.09)	0.14 (0.04–0.27)	3.33 (2.22–3.89)^°^^#^	0.07 (0.00–0.57)

D_s_, dietary intake, screening. D_wk12_, dietary intake at wk 12. T_wk12_, total intake at wk 12. P < 0.05^*^.

° *Significantly different compared to D_s_*;

# *Significantly different compared to D_wk12_. P-value between 3 moments: before breakfast (p = 0.513), breakfast (p ≤ 0.001), snack 1 (p = 0.589), lunch (p = 0.589), snack 2 (p = 0.819), dinner (p = 0.010), and snack 3 (p = 0.106)*.

*Bold p-values represent statistically significant results (p < 0.05)*.

At screening, the regular dietary distribution of leucine intake at breakfast, lunch, dinner and snack times was unequal, and no meal reached the 2.5 g leucine threshold (Table 4). Protein supplementation resulted in higher total leucine intake (2.46 g at breakfast, 3.33 g at dinner) compared to regular dietary intake at screening at breakfast (0.87 g, *p* < 0.001) and dinner (1.99 g, *p* = 0.009) or compared to regular dietary intake at wk 12 at breakfast (0.90 g, *p* < 0.001) and dinner (2.35 g, *p* < 0.001; Table 4).

### Compliance

**Figure 3:**
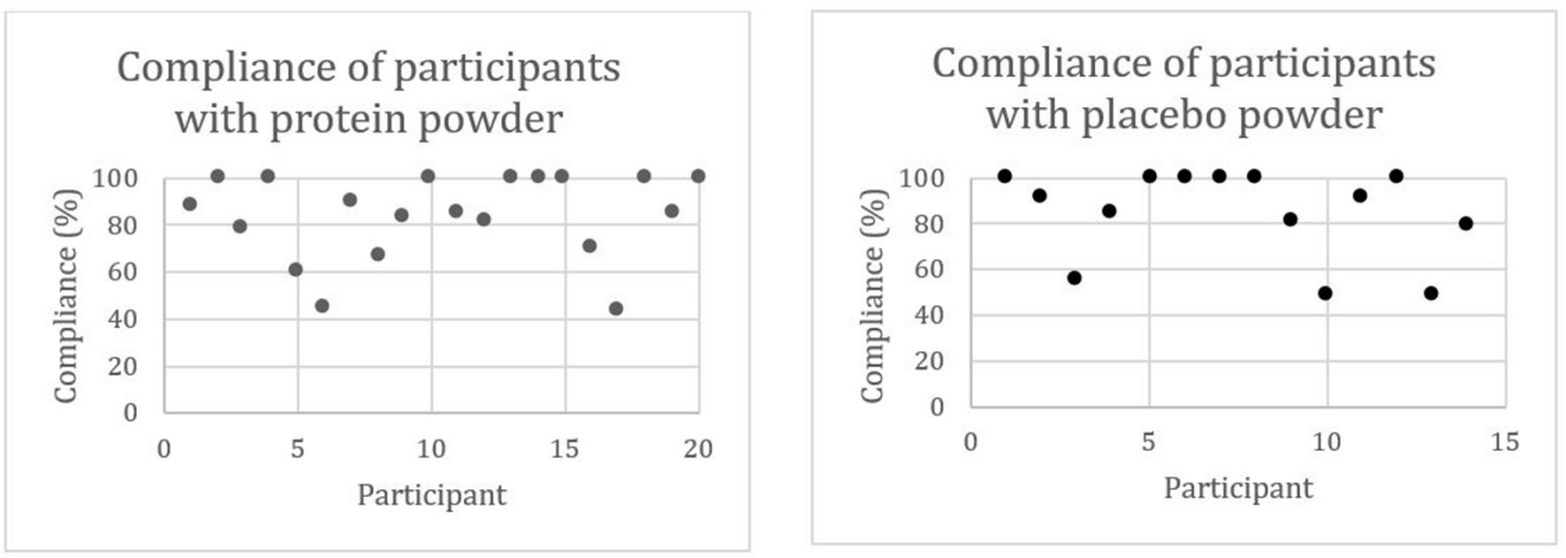
Compliance (%) of community-dwelling (pre)sarcopenic older adults with protein (*n* = 20) or placebo (*n* = 15) powder intake.

The compliance with protein powder intake ranged from 44 to 100%, and the compliance with placebo powder intake ranged from 49 to 100%. Seventy-five percent (15/20) of the participants with protein powder supplementation and 79% (11/14) of the participants with placebo powder supplementation had a compliance higher than 79%. Compliance of one participant of the placebo powder supplementation group was not calculated as this person did not bring the boxes to the study visits. Figure 3 visualizes the compliance on an individual level.

## Discussion

The present study showed that the quantity, quality and distribution of regular dietary protein or leucine intake of community-dwelling (pre)sarcopenic older adults do not reach the recommended levels (16). However, we showed that the addition of a personalized protein supplement resulted in an adequate quantity and quality total protein intake, and a more even distribution over the day, without affecting the protein or energy intake through the diet.

Regular dietary protein intake in Dutch older individuals ranged from 0.78 ± 0.3 g·kg^−1^·d^−1^ of body mass in institutionalized older men to 1.11 ± 0.3 g·kg^−1^·d^−1^ of body mass in community-dwelling older men (19). Bollwein et al. (18) reported a regular dietary protein intake of 1.07 (0.6–2.3) g·kg^−1^·d^−1^ of body mass in German community-dwelling older persons (18). Regular dietary protein intake in (pre)sarcopenic community-dwelling older adults described in this study is in line with these previous studies. Discussing this result is not straightforward, as there is no consensus yet on the “optimal” protein intake of older adults. The World Health Organization (WHO) RDA of 0.8 g·kg^−1^·d^−1^ of body mass is based on 19 nitrogen balance studies, only one of which has been performed in older adults. Moreover, the NB technique is now considered inappropriate for making recommendations (37, 38), and is replaced by the indicator amino acid oxidation (IAAO) method. Experiments with this latter technique in older adults (>65 years) resulted in higher RDA values: 1.29 g·kg^−1^·d^−1^ of body mass for women and 1.24 g·kg^−1^·d^−1^ of body mass for men (39, 40). However, protein intake that aims to reach the RDA, which aims to avoid deficiencies, is not equal to optimizing clinical outcomes. Protein intake in accordance with the RDA may not always lead to optimally stimulated MPS (41). Other studies examined the effect of a protein dose higher than the RDA. First, compared to a single dose RDA (0.8 g·kg^−1^·d^−1^ of body mass), a double dose RDA (1.5 g·kg^−1^·d^−1^ of body mass) resulted in increased MPS and whole-body lean mass, maintenance of lean mass and less decrease in appendicular lean mass and leg power (42–44). Second, increasing the protein intake from 0.8 g·kg^−1^·d^−1^ of body mass to 1.2 g·kg^−1^·d^−1^ of body mass showed only limited effects on MPS (45). Taken together, it is expected, that optimal protein dose for older adults is higher than the current RDA as proposed by the WHO (38).

In our study in (pre)sarcopenic older adults, regular dietary protein intake was unevenly distributed over the day, with the highest protein intake at lunch and the lowest at breakfast. These results are similar to the protein intake distribution in a Dutch community-dwelling, frail or institutionalized population (19) or a German frail population (18). Also in a French older population, protein was mainly consumed at lunch (56.5% of all proteins) (46). However, in other countries such as Norway, protein intake was highest at dinner in older adults (47). Belgian older adults commonly consume a warm meal for lunch, which is associated with a higher protein intake compared to bread-based meals (19).

The aim of this study was not only to achieve adequate levels of total protein intake per day, but also to achieve an even distribution of 25–30 g protein per meal. Indeed, reviews suggest an optimal MPS with evenly distributed protein of 25–30 g·meal^−1^ (13, 16), or 30–40 g·meal^−1^ (12), or state that there is no practical upper limit to the anabolic response to protein intake in the context of a meal (48). No upper limit is observed when also the rate of protein breakdown is taken into account. Higher protein intake results in greater insulin response with subsequent suppression of protein breakdown and thus a greater anabolic response (48). Protein supplementation could improve the distribution of protein intake in (pre)sarcopenic older adults throughout the different meals.

Regarding the factors that influence protein quality, previous research showed that animal-based proteins stimulate MPS more than plant-based proteins, which is caused by, among others, a higher relative leucine content (49). Our study showed highest contribution of animal proteins to the total protein intake, which is in line with Dutch community-dwelling, frail or institutionalized older adults (65% from animal origin) (19) and American adults over 71 years of age (61% from animal origin) (50). Also, our study showed that the leucine intake was approximately 114 mg·kg^−1^·d^−1^ of body mass. This is higher than the assumed optimal 55 mg·kg^−1^·d^−1^ of body mass for healthy adults (51), but lower than in older (>70 years) men (160 mg) and women (130 mg) in the National Health and Nutrition Examination Survey III study (NHANES) (52). Moreover, the distribution of leucine in our study sample was uneven and low at breakfast and dinner. A study in healthy older adults without functional limitations showed a more even distribution (1.97 ± 1.31 g at breakfast, 2.5 ± 1.27 g at lunch, and 2.27 ± 1.26 g at dinner) (53). After protein supplementation, the leucine intake and distribution, and thus the dietary protein quality, was improved without affecting regular dietary protein or energy intake. To the best of our knowledge is this the first study assessing leucine distribution in (pre)sarcopenic community-dwelling older adults. We also found a slight increase in dietary cysteine intake at wk 12, however, it is unclear if this difference also is clinically relevant. The WHO defines the total sulfur amino-acid requirement (cysteine and methionine) as 15 mg/kg·day^−1^ (54). Our study showed a median sulfur amino-acid intake after supplementation of 3.27 g·day^−1^ (cysteine (1.23g·day^−1^) and methionine (2.04g·day^−1^), which is equal to 54.5 mg/kg·day^−1^ for a person weighing 60 kg.

The contribution of protein to the daily energy intake was similar in our study (15.8%) as in similar study samples in Germany [15.9% (18)] and the USA [15.4% (50)]. Also the total daily energy intake is similar among the participants of our study (1,830 ± 344 kcal·day^−1^) compared to prefrail (1,800 ± 636 kcal·day^−1^) and robust (1,869 ± 652 kcal·day^−1^) community-dwelling older adults of the Health ABC study (55) or women (1,464 kcal·day^−1^) and men (2,034 kcal·day^−1^) over 71 years of age in the National Health and Nutrition Examination Survey study (50). Higher intake was reported in Dutch older men in the community (to 2,246 ± 573 kcal·day^−1^), and lower intake in Dutch older men in institutions (1,385 ± 358 kcal·day^−1^) (19). Maintaining energy levels is important since higher energy intake is associated with lower hazard ratios of both all-cause and cardiovascular disease mortality in older adults (56).

The main strength of our study is that the participant's protein supplementation is personalized, in contrast to other studies that supplement a certain fixed amount of protein, for example 20 g per meal (57). Moreover, this study indicated that 12 weeks supplementing with a personalized powder supplement seems possible in this population, which is not achievable in all types of populations (58). Another strength of our study is the use of the EDR. The participants are asked to record all foods and beverages at the time it occurs, to minimize dependence on memory. Although the participant's reactivity behavior may affect the EDR (30), it was shown that food records provide better estimates of protein and energy intake compared to 24-h dietary recalls and a food frequency questionnaire (59). Moreover, the compliance with the supplement intake was assessed by weighing the amount of powder that the participant returned to the study visit. It must therefore be reported that this measurement is only an indication of compliance and not a measure of effective consumption.

One limitation of this study is the limited number of participants who received the protein supplement (*n* = 20). The participants represent a range of sarcopenia stages. A relative high number of participants dropped-out after randomization. As a result of these three points, the representability of this study sample may be implicated and potentially limits the external validity of the proof of concept. Concerning the methodology, a few topics can be discussed. First, protein supplementation focused on the main meals, whilst snack 3 (before bedtime) was only supplemented if the main meals could insufficiently be increased. It could be argued that protein intake before bedtime could have been included to increase the fractional synthetic rate during overnight recovery (60). Second, the analysis of the amino-acid composition of the dietary protein intake in this study mainly focuses on leucine, given its role in the development of muscle tissue MPS in older adults (61). However, since the stimulation of MPS by leucine also depends on the availability of other amino acids (62), these could also be discussed in light of their recommended amount in future studies. Third, the use of the EDR is the best available method to assess protein intake for the purpose of this study, but the validity of this technique can be questioned since it is based on estimates and not quantitative measures. Fourth, the effect of other interventions such as exercise or PUFA's supplementation was not taken into account for the analysis of the regular dietary intake or the effect of supplementation on regular dietary intake nor did we assess effect of the different interventions on sarcopenia status and functional outcomes. Recent studies found that sarcopenia was more frequent in community-dwelling adults with low levels of leucine and essential amino acids in the blood (63), and associated with decreased consumption of protein compared to non-sarcopenic adults (64). This was not assessed in this study yet can be a focus for further research.

### Means for Practice

The results of this study showed that personalized protein supplementation is needed to achieve recommended dietary protein intake, and showed to be achievable to improve protein quantity, quality and distribution in community-dwelling (pre)sarcopenic older adults (16). Since diet is a modifiable risk factor for sarcopenia (65), influencing nutritional status may influence muscle functioning and therefore for example sarcopenia, a syndrome that increases the health care expenditures with $900/person/year (66). In that respect, nutritional adjustments can be considered as relatively inexpensive interventions, for example by dieticians. Consequently, the effect of personalized protein supplementation on outcomes such as sarcopenia or physical functioning should be one of the focal points of further research.

## Data Availability Statement

The raw data supporting the conclusions of this article will be made available by the authors, without undue reservation.

## Ethics Statement

The studies involving human participants were reviewed and approved by The study was approved by KU Leuven/UZ Leuven Ethics Committee (s60763). The patients/participants provided their written informed consent to participate in this study.

## Author Contributions

LD, SV, KK, JT, and EG: conceptualization. LD and JD: data curation. LD and JD: investigation. LD, JD, JT, CM, and EG: methodology. EG: supervision. LD and EG: writing – original draft. LD, JD, SV, KK, JT, CM, and EG: writing – review and editing. All authors contributed to the article and approved the submitted version. A version of this article previously appeared online in the PhD thesis of LD (67).

## Conflict of Interest

JD received a fellowship grant (11A9320N) from Research Foundation Flanders (FWO). JT: speaker's fees from Nutricia. EG: consultancies/advisory boards and/or speaker's fees from Amgen, Sandoz, Takeda, UCB. The remaining authors declare that the research was conducted in the absence of any commercial or financial relationships that could be construed as a potential conflict of interest.

## Publisher's Note

All claims expressed in this article are solely those of the authors and do not necessarily represent those of their affiliated organizations, or those of the publisher, the editors and the reviewers. Any product that may be evaluated in this article, or claim that may be made by its manufacturer, is not guaranteed or endorsed by the publisher.

## Supplementary Material

Supplementary Figure 1 Overview of assessment moments in the ENHANce study relevant for this study. The moments at which the data was collected for the use in this study are indicated with an orange arrow. EDR, estimated dietary records. Symbols from https://icon-library.net.

Supplementary Figure 2 Overview of individual protein supplementation (g) per meal moment (*n* = 20).

Supplementary Table 1 Nutritional content of protein and placebo supplement.

Supplementary Table 2 Distribution of protein intake (median and interquartile range) based on the four-day EDR of all and subgroup of 20 community-dwelling pre)sarcopenic older adults receiving protein supplement.

Supplementary Table 3 Total intake of amino acids (g) per day (median and interquartile range) of subgroup of 20 community-dwelling (pre)sarcopenic older adults receiving protein supplement.

## Abbreviations

BMI: body mass index
EDR: estimated dietary records
ENHANce: exercise and nutrition for healthy aging
EWGSOP: European working group on sarcopenia in older people
MNA-SF: Mini nutritional assessment—short form
MPS: muscle protein synthesis
RDA: recommended dietary allowance
SMI: Skeletal muscle index
Wk 12: week twelve
WHO: World health organization.
