# Supplementary figures and images for: Personalized Protein Supplementation Improves Total Protein, Leucine, and Energy Intake in (Pre)Sarcopenic Community-Dwelling Older Adults in the ENHANce RCT
Source: Front Nutr. 2021 Aug 9;8:672971. doi: 10.3389/fnut.2021.672971 (PMC8381276; doi:10.3389/fnut.2021.672971)

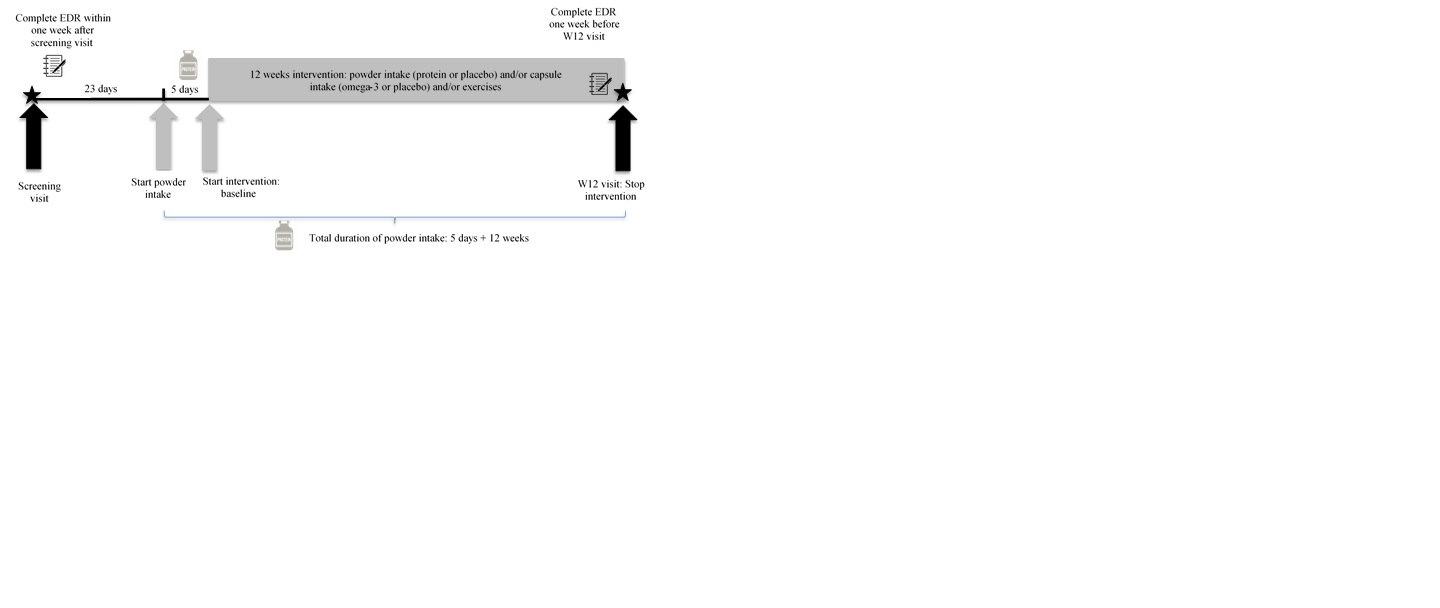

Supplement: Supplementary Figure 1 — Overview of assessment moments in the ENHANce study relevant for this study. The moments at which the data was collected for the use in this study are indicated with an orange arrow. EDR, estimated dietary records. Symbols from https://icon-library.net. [file Image_1.JPEG]

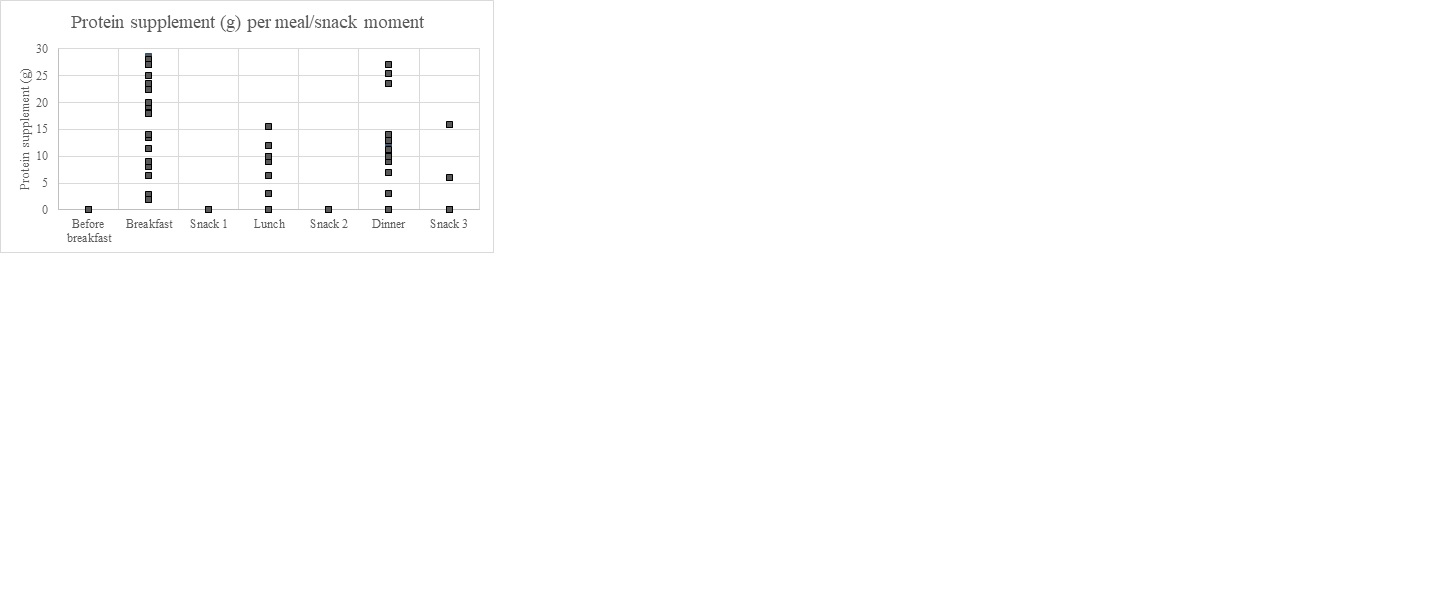

Supplement: Supplementary Figure 2 — Overview of individual protein supplementation (g) per meal moment (n = 20). [file Image_2.JPEG]
