# Supplementary material for: Personalized Protein Supplementation Improves Total Protein, Leucine, and Energy Intake in (Pre)Sarcopenic Community-Dwelling Older Adults in the ENHANce RCT
Source: Front Nutr. 2021 Aug 9;8:672971. doi: 10.3389/fnut.2021.672971 (PMC8381276; doi:10.3389/fnut.2021.672971)
Supplement: Supplementary Table 1 — Nutritional content of protein and placebo supplement. [file Table_1.DOCX]

Supplementary Material

# Supplementary data

# Supplementary Table 1: Nutritional content of protein and placebo supplement

|  | Protein supplement, 100 g | Placebo supplement, 100 g |
| --- | --- | --- |
| Energy, kcal | 371 | 381 |
| Proteins (of which leucine, %) g | 90 (9.14%) | 0.2 (0%) |
| Carbohydrates, g | 0.50 | 95 |
| Fat, g | 1.0 | 0.0 |
