# Supplementary material for: Personalized Protein Supplementation Improves Total Protein, Leucine, and Energy Intake in (Pre)Sarcopenic Community-Dwelling Older Adults in the ENHANce RCT
Source: Front Nutr. 2021 Aug 9;8:672971. doi: 10.3389/fnut.2021.672971 (PMC8381276; doi:10.3389/fnut.2021.672971)
Supplement: Supplementary Table 2 — Distribution of protein intake (median and interquartile range) based on the four-day EDR of all and subgroup of 20 community-dwelling pre)sarcopenic older adults receiving protein supplement. [file Data_Sheet_1.PDF]

Supplementary Material

1. Supplementary data

Supplementary Table 2: Distribution of protein intake (median and interquartile range) based on the four-day EDR of all and subgroup of 20 community-dwelling pre)sarcopenic older adults receiving protein supplement.

	Before breakfast	Breakfast	Snack 1	Lunch	Snack 2	Dinner	Snack 3
**Screened participants (*n* = 51)**							
**Dietary intake, screening (g·meal^{-1})**	0.00 (0.00 - 0.24)	10.9 (7.09 – 16.3)	0.26 (0.00 – 0.94)	28.1 (21.3 – 37.7)	1.77 (0.78 – 2.99)	25.3 (17.7 – 34.0)	1.57 (0.36 – 3.86)
**Protein (*n* = 20)**							
**Dietary intake, screening (g·meal^{-1})**	0.00 (0.00 - 0.58)	11.7 (7.12 – 14.6)	0.28 (0.06 – 0.79)	26.2 (21.9 – 33.9)	1.34 (0.71 – 2.15)	24.4 (17.1 – 32.3)	1.82 (0.32 – 3.95)
**Dietary intake, wk 12 (g·meal^{-1})**	0.00 (0.00 - 0.36)	10.2 (7.07 – 16.7)	0.37 (0.00 - 2.36)	24.7 (18.9 – 33.2)	1.145 (0.94 – 2.80)	26.2 (16.4 – 37.2)	1.06 (0.00 – 2.77)°
**Total intake, wk 12 (g·meal^{-1})**	0.00 (0.00 - 0.36)	28.2 (22.5 – 34.8)°#	0.37 (0.00 - 2.36)	31.3 (23.2 – 35.8)	1.45 (0.94 – 2.80)	36.7 (26.3 – 45.9)° #	1.06 (0.00 – 4.64)

D_s: dietary intake, screening. D_{wk12}: dietary intake at wk 12. T_{wk12}: total intake at wk 12. ° : significantly different compared to D_s; # : significantly different compared to D_{wk12}. *P*-value between 3 moments: before breakfast (p = 0.819), breakfast (p = <0.001), snack 1 (p = 0.589), lunch (p = 0.161), snack 2 (p = 0.449), dinner (p = 0.004), snack 3 (p = 0.044).
