# Supplementary material for: Personalized Protein Supplementation Improves Total Protein, Leucine, and Energy Intake in (Pre)Sarcopenic Community-Dwelling Older Adults in the ENHANce RCT
Source: Front Nutr. 2021 Aug 9;8:672971. doi: 10.3389/fnut.2021.672971 (PMC8381276; doi:10.3389/fnut.2021.672971)
Supplement: Supplementary Table 3 — Total intake of amino acids (g) per day (median and interquartile range) of subgroup of 20 community-dwelling (pre)sarcopenic older adults receiving protein supplement. [file Data_Sheet_2.PDF]

Supplementary Material

1. Supplementary data

Supplementary table 3: Total intake of amino acids (g) per day (median and interquartile range) of subgroup of 20 community-dwelling (pre)sarcopenic older adults receiving protein supplement.

**Amino-Acid**	**Screening (g·day^{-1})**	**Week 12 (only dietary intake) (g·day^{-1})**	***P*-value**
**Leucine**	6.39 (5.20 – 7.07)	7.03 (5.68 – 7.93)	0.232
**Isoleucine**	3.57 (2.92 – 4.14)	4.06 (3.24 – 4.43)	0.313
**Valine**	4.32 (3.61 – 4.95)	4.79 (3.80 – 5.47)	0.218
**Lysine**	5.57 (4.64 – 6.15)	6.07 (4.92 – 7.10)	0.313
**Methionine**	1.88 (1.50 – 2.02)	2.04 (1.60 – 2.27)	0.313
**Phenylalanine**	3.56 (2.92 – 3.85)	4.02 (3.12 – 4.38	0.218
**Tryptophan**	0.97 (0.74 – 1.08)	1.00 (0.83 – 1.12)	0.478
**Threonine**	3.13 (2.50 – 3.52)	3.55 (2.74 – 4.01)	0.204
**Histidine**	2.34 (1.90 – 2.59)	2.50 (1.90 – 2.98)	0.332
**Arginine**	3.85 (3.43 – 4.30)	4.38 (3.36 – 5.23)	0.232
**Cysteine**	1.05 (0.95 – 1.20)	1.23 (1.00 – 1.37)	**0.048**
**Glycine**	3.57 (2.83 – 4.29)	3.70 (3.49 – 4.46)	0.370

**Proline**	5.55 (4.72 – 6.14)	6.30 (5.11 – 7.10)	0.156
**Tyrosine**	2.51 (2.03 – 2.86)	2.63 (2.18 – 3.30)	0.313
**Alanine**	3.68 (2.86 – 4.00)	3.99 (3.12 – 4.46)	0.247
**Aspartic acid**	7.07 (5.71 – 7.80)	7.69 (5.63 – 8.76)	0.433
**Glutamic Acid**	15.5 (12.1 – 17.3)	16.7 (13.9 – 19.1)	0.247
**Serine**	3.72 (3.07 – 3.93)	4.07 (3.26 – 4.81)	0.156
